# A Comparative Study on the Sensitivity of Establishing Melasma‐Like Models in Different Strains of Mice

**DOI:** 10.1111/jocd.70155

**Published:** 2025-03-31

**Authors:** Xiaojie Sun, Wenzhu Wang, Hedan Yang, Yunyao Liu, Hui Ding, Xiaoli Zhang, Xiuzhen Li, Siqi Tan, Xing Liu, Yin Yang, Xu Chen, Tong Lin

**Affiliations:** ^1^ Department of Laser, Hospital for Skin Diseases, Institute of Dermatology Chinese Academy of Medical Sciences & Peking Union Medical College Nanjing China; ^2^ Jiangsu Key Laboratory of Molecular Biology for Skin Diseases and STIs Department, Hospital for Skin Diseases, Institute of Dermatology Chinese Academy of Medical Sciences & Peking Union Medical College Nanjing China

**Keywords:** BALB/c mice, C57BL/6J mice, KM mice, melasma, mouse model

## Abstract

**Background:**

Melasma is a common disease that is difficult to treat, with no recognized animal model for mechanism research or drug screening.

**Objectives:**

To develop a novel, standardized mouse model of melasma.

**Materials and Methods:**

Three different strains of mice (C57BL/6J, BALB/c, and KM) were used to create a melasma‐like model across various body regions. The skin of the mice was removed on Day 28 and subjected to staining to examine histopathological changes. Data were analyzed using SPSS19.0 software.

**Results:**

Compared with KM and BALB/c mice, C57BL/6J mice were identified as the ideal strain for demonstrating hyperpigmentation more sensitively. The head and ear were identified as more appropriate irradiation sites. Furthermore, a lower irradiation dose was determined to be appropriate for modeling.

**Conclusion:**

The C57BL/6J mouse model more accurately simulates the clinical phenotype of melasma.

## 1 Introduction

Melasma is a common, acquired pigmentary disease of the skin, mainly manifesting as brown patches symmetrically distributed on the cheeks, forehead, and jaw with unclear borders [1, 2]. It has been reported that more than 5 million patients suffer from melasma in the United States. The prevalence of melasma in the Asian population has been noted to reach as high as 20.5%–33%, predominantly affecting women of reproductive age [1, 2, 3]. Melasma primarily occurs on the face, hindering patients' social interactions. Patients with melasma often experience depression, low self‐esteem, and other negative emotions, which severely affect their physical and mental health as well as overall quality of life [4].

Developing an appropriate animal model of melasma is invaluable for advancing melasma research. Choosing an appropriate animal model helps to further elucidate melasma pathogenesis and facilitates the study of effective treatments and prevention methods. Currently, there is no universally recognized method for developing melasma animal models. Although China has issued draft guidelines for preparing melasma‐like animal models [5], key factors such as specific strains and exposure doses have not been detailed. Reported animal models used in melasma research include guinea pigs, SD rats, C57BL/6J mice, BALB/c mice, KM mice, and HRM‐2 hairless mice [6, 7, 8, 9, 10]. Due to their convenience for genetic editing and further research on molecular mechanisms, C57BL/6J, BALB/c, and KM mice were employed to establish a melasma‐like mouse model that more closely mimics the clinical features of the condition. The skin appearance and histopathology of the melasma model in different strains and regions of the mice were compared to determine which strain and body region were most suitable and easier for establishing a melasma‐like mouse model. The main purpose of this study is to provide an appropriate mouse model for studying the molecular mechanisms of melasma.

## 2 Materials and Methods

### 2.1 Animals

**FIGURE 1:**
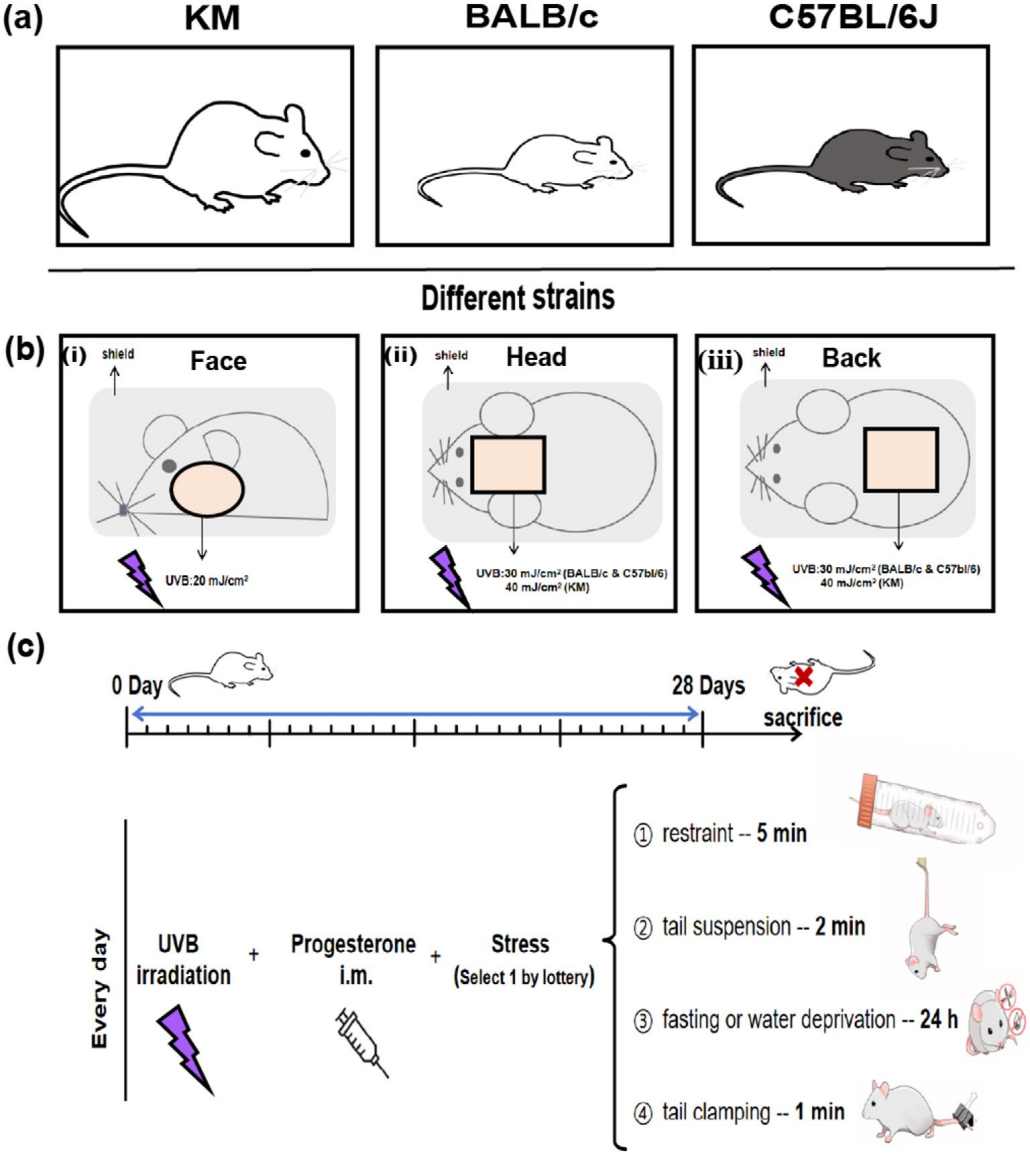
The method of creating melasma‐like animal model in mice. (a) Animal strains: KM mice, BALB/c mice and C57BL/6 mice were used to compare the skin changes of different strains of mice in creating melasma‐like mouse model. (b) All three strains of mice were divided into three groups according to different sites to model. (i) Three face groups of different strains were exposed to UVB with a dose of 20 mJ/cm^2^; (ii) head groups of BALB/c mice and C57BL/6 mice were exposed to UVB with a dose of 30 mJ/cm^2^ (40 mJ/cm^2^ for KM mice); (iii) back groups of BALB/c mice and C57BL/6 mice were exposed to UVB with a dose of 30 mJ/cm^2^ (40 mJ/cm^2^ for KM mice). Areas outside the irradiation area were covered with tin foil, *N* = 5 for each group. (c)Time‐flow chart of the melasma‐like mouse model.

As shown in Figure 1a, healthy 6‐ to 8‐week‐old nonpregnant, SPF BALB/c and C57BL/6J mice, weighing (20 ± 2) g, and SPF KM mice, weighing (35 ± 2) g, were purchased from Jiangsu Jicuiyaokang Biotechnology Co. Ltd. All mice were raised in accordance with the animal experimental guidelines of our institution. In the SPF animal feeding room, the temperature was controlled at (22 ± 2)°C, the humidity was maintained between 60% and 70%, and a 12‐h light/dark cycle was maintained. All the animal experiments were approved by the animal ethics committee of our institution and conformed to the existing current animal welfare guidelines.

### 2.2 Main Reagents and Instruments

Mice were exposed to ultraviolet radiation b (UVB) using a broadband UVB lamp (Philips, The Netherlands), which emitted wavelengths between 290 and 320 nm. The radiation intensity was maintained at 2.8 mW/cm^2^, as measured by a UV radiometer. Mice were irradiated for 7–33 s at a distance of 15 cm, receiving a total dose of 20–90 mJ/cm^2^. Additionally, mice were injected into the hind limb muscle with progesterone injection solution (Tianjin Jinyao Pharmaceutical Co. Ltd., National Drug Standard H12020534). Some mice were intraperitoneally injected with tranexamic acid (TXA) solution (B34539, Shanghai Yuanye Biotechnology Co. Ltd.).

### 2.3 Methods

The mice were housed adaptively for 3 days. At the end of the adaptation period, five mice in each group were randomly divided into BALB/c‐face, BALB/c‐head, BALB/c‐back, C57BL/6J‐face, C57BL/6J‐head, C57BL/6J‐back, C57BL/6J‐ear (60 mJ/cm^2^), C57BL/6J‐ear (90 mJ/cm^2^), KM‐face, KM‐head, and KM‐back. One day prior to modeling, the hair of the model was removed using an electric razor, and a mild depilatory cream was applied to remove hair from the corresponding area of the mouse. Prior to irradiation, photographs were taken to document the condition of the modeled area. In the course of continuous irradiation for 28 days, each mouse was depilated according to the hair growth of the mice, and the frequency was once every 5–7 days, in which the shaving time could be appropriately shortened to 2–3 days during the hair cycle. Do the following three ways everyday and take photos on the 28th day. Then the mice were euthanized by CO_2_ inhalation, and the corresponding tissues were collected for follow‐up experiments. For the mice treated with this combination of ultraviolet irradiation, progesterone injection, and creating emotional stress as the model group, mice treated with additional TXA intervention are defined as the TXA group.

#### 2.3.1 Ultraviolet Irradiation

In the face group, the hair was shaved on both sides of the face, and the skin in the shaving area was locally exposed to a dose of 20 mJ/cm^2^. In the head group, the hair was shaved between the ears (1 × 1 cm^2^), and the skin in the shaving area was locally exposed to a dose of 30 mJ/cm^2^ (KM is 40 mJ/cm^2^). In the back group, the hair was shaved on the middle back (1.5 × 1.5 cm^2^), and the skin in the shaving area was locally exposed to a dose of 30 mJ/cm^2^ (KM is 40 mJ/cm^2^). In the ear group, only the left ear was irradiated with a dose of 60, 90 mJ/cm^2^. The unirradiated area is covered with tin foil (Figure 1b). All the doses of ultraviolet irradiation were according to the minimal erythema dose (MED). During the entire experiment, the mice were under inhalation anesthesia.

#### 2.3.2 Progesterone Injection

After irradiation, 0.4 mg/kg (5 mL/kg) progesterone was administered via injection into the hind limb muscle of the mice once daily, alternating between the two hind limbs.

#### 2.3.3 Create Emotional Stress

Choose one of the following ways every day by lottery (Randomly arrange each way within each week, but only 1 day of fasting or water deprivation. Besides, keep consistent processing sequence between different batches). (i) Restraint: several holes were made in a 50‐mL plastic tube to maintain airflow, and the animal was placed in this tube for 5 min, thereby exposing it to restraint stress; (ii) tail suspension: The tail of the mouse was secured during the suspension process for 2 min each time; (iii) fasting or water deprivation for 24 h; (iv) tail clamping: The tail pincher was placed 2 cm from the tail end for 1 min with moderate force so as not to injure the tail of the mouse (Figure 1c).

#### 2.3.4 TXA Injection

For the mice in the TXA group, after the treatment mentioned above, 65 mg/kg/day TXA solution was intraperitoneally injected.

### 2.4 Histological Analysis

Skin tissues were fixed in 10% neutral formalin tissue fixation solution for 24 h, dehydrated, and embedded in paraffin. The paraffin sections of skin (both human and mouse) were made for hematoxylin and eosin staining (H&E staining).

### 2.5 Masson‐Fontana Staining

Melanin of the skin tissues was detected with Masson‐Fontana staining. Then the slices of paraffin‐embedded tissue were observed under a light microscope and photographed with a final magnification of 20× and 40×. Image analysis was conducted using ImageJ software (version 1.53a, National Institutes of Health, Bethesda, MD, USA) to quantify the percentage of pigmented area relative to the total epidermal and dermal area (area %).

### 2.6 Melan A and SOX 10 Staining

Melanocytes in the skin tissues were detected using Melan A and SOX 10 staining. The samples were stained with anti‐SOX 10 (SD204‐04, HUABIO) and anti‐Melan A monoclonal antibody (ab210546, Abcam). The paraffin‐embedded tissue sections were then observed under a light microscope and photographed at a final magnification of 20×.

### 2.7 Population

Patients aged ≥ 18 years old with melasma clinically diagnosed under Wood's light examination were included. The study excluded patients with another pigmentation disorder on the face, having received any depigmenting cosmetic, systemic drugs, topical hydroquinone, or retinoic acid on the face within 1 month prior to inclusion. Patients being pregnant and breastfeeding women, or planning to have a child during the 24 weeks of the study, were excluded. All patients provided informed consent.

### 2.8 Skin Lesion of Human

One adult woman with facial melasma underwent a biopsy (2‐mm punch, using a sterile technique) of the melasma skin lesion and the adjacent region of clinically normal skin lesion(< 2 cm of distance). The participant was untreated for the dermatosis for at least 30 days. The study was approved by the ethics committee of our institution.

### 2.9 In Vivo Reflectance Confocal Microscopy (RCM) Examination

We used a commercially available RCM apparatus (Vivascope 1500; Lucid Inc) for skin imaging. The RCM provided distinct images of the cellular structures of the examined lesions in vivo with high optical resolution. Each image showed a horizontal 500 × 500‐μm section at an adjusted depth from the epidermal surface to the dermis. Twenty‐six adult women with facial melasma were involved in this experiment, and all patients signed an informed consent.

### 2.10 Statistical Analysis

The melanin staining content was analyzed using SPSS 19.0 software and expressed as the mean ± standard deviation (x̄ ± SD). Statistical significance was assessed using a *t*‐test and one‐way ANOVA (*p* < 0.05).

## 3 Results

### 3.1 General Observation and Histological Manifestations of Normal Skin in KM, BALB/c, and C57BL/6J Mice

**FIGURE 2:**
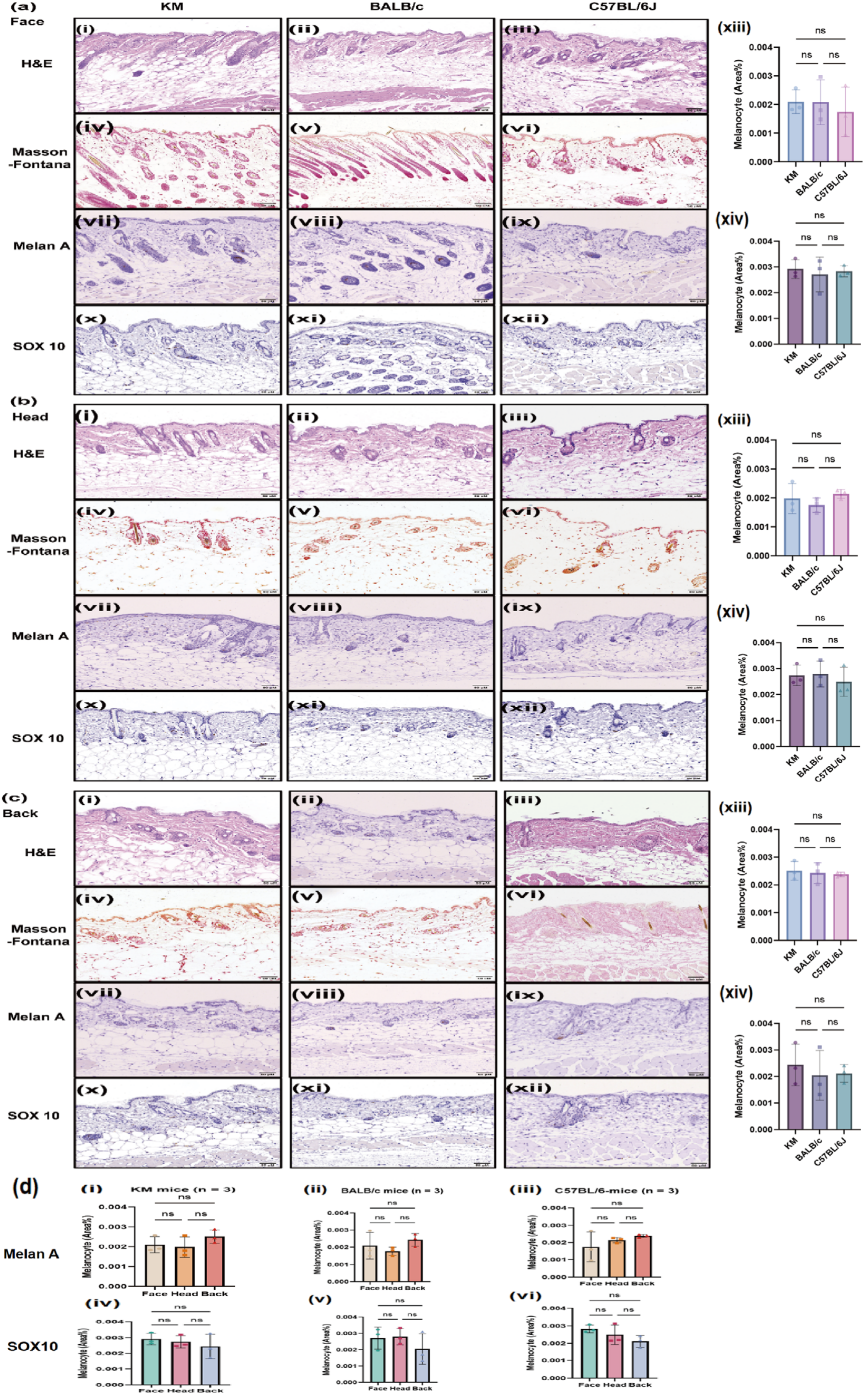
General observation and histological manifestations of normal skin in KM, BALB/c and C57BL/6J mice. (a) Face group. (b) Head group. (c) Back group. (i–iii) H&E staining results of different strains; (iv–vi) Masson‐Fontana staining results of different strains; (vii–ix) Melan A staining results of different strains; (x–xii) SOX 10 staining results of different strains; (xiii) statistical analysis results of Melan A staining in different strains of mice; (xiv) statistical analysis results of SOX 10 staining in different strains of mice; (d) statistical analysis results of Melan A and SOX 10 staining in different regions of mice. *N* = 3 for each group, scale bar = 50 μM.

KM, BALB/c, and C57BL/6J mice were selected as previously reported [8, 9, 10], and various body regions were chosen to compare histological characteristics. H&E staining revealed that, within the same mouse species, the dermis on the facial surface was the thinnest, while the back was the thickest. The density of dermal follicle appendages was highest on the face, while the back showed a density similar to that of the head (Figure 2). Masson‐Fontana staining, a common method for evaluating melanin distribution, revealed melanin granules in the dermis of C57BL/6J mice, but not in KM or BALB/c mice (Figure 2). Additionally, immunohistochemical staining for Melan A and SOX 10 was used to detect melanocytes in all strains of mice, demonstrating that melanocytes are primarily distributed in the hair follicles of the skin (Figure 2).

### 3.2 General Observation and Histological Manifestations of Melasma‐Like Skin Lesions in KM and BALB/c Mice

**FIGURE 3:**
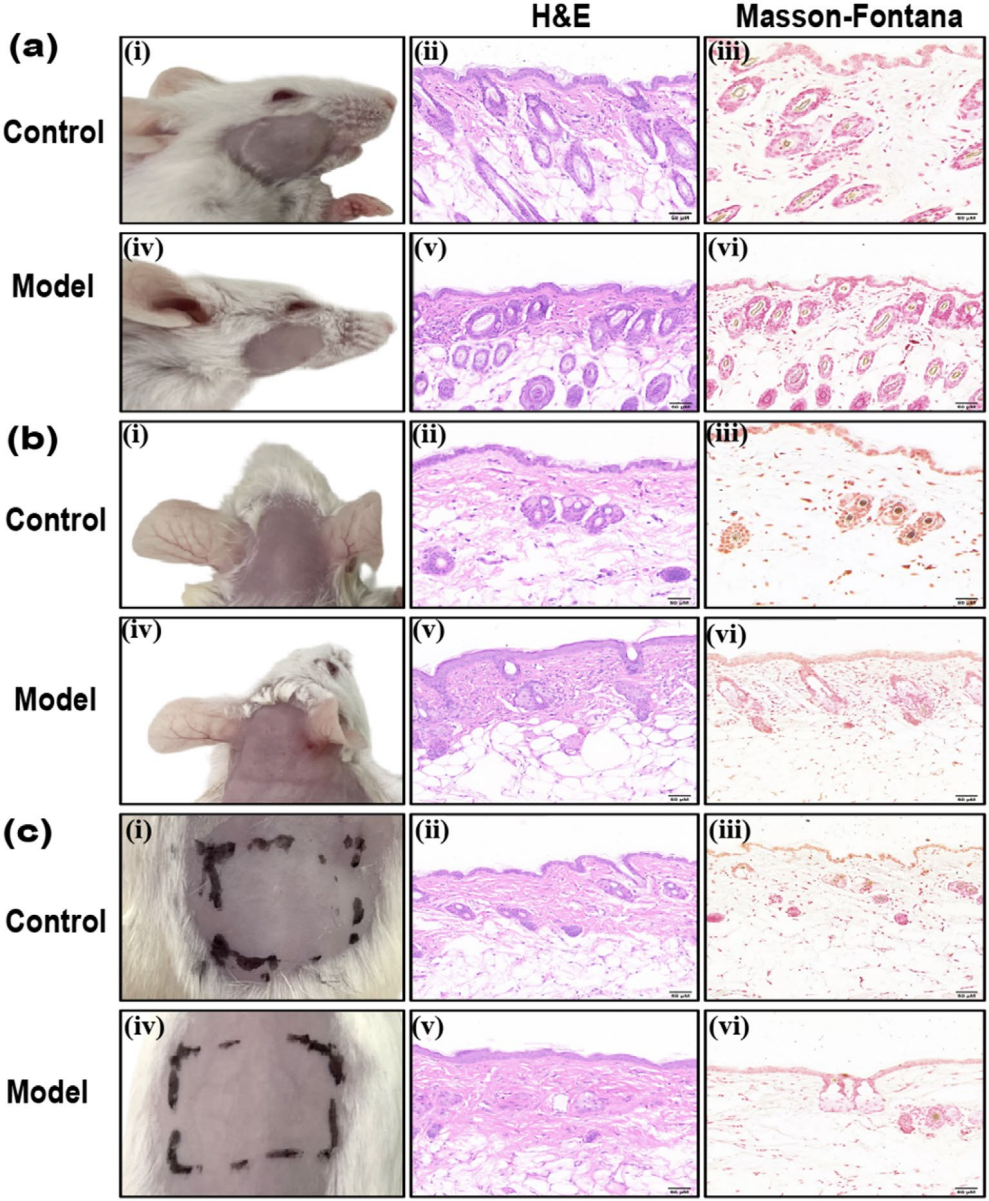
Clinical observation and histopathological manifestations of melasma‐like skin lesions in KM mice. (a) Face group. (b) Head group. (c) Back group. *N* = 5 for each group, scale bar = 50 μM.

**FIGURE 4:**
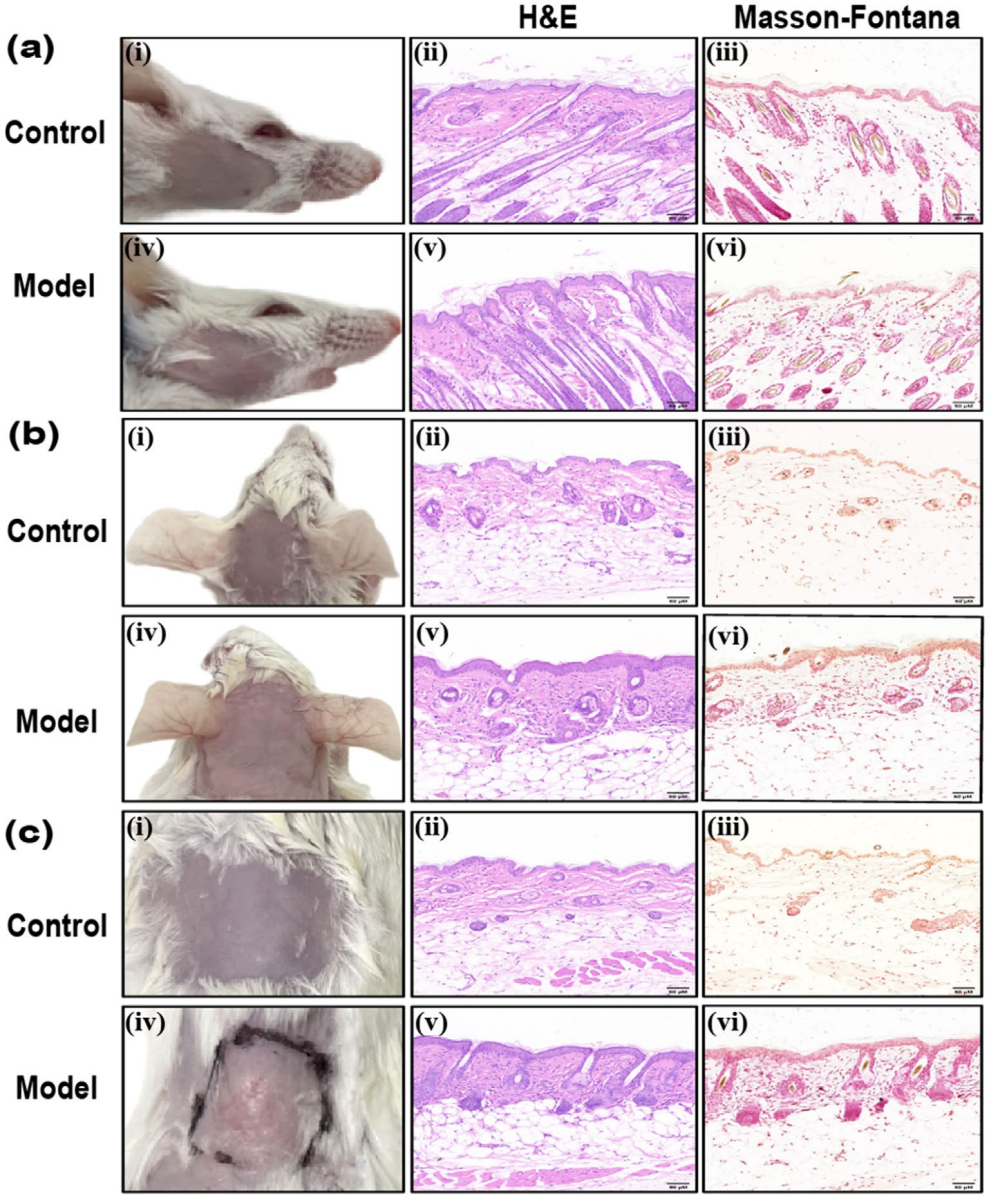
Clinical observation and histopathological manifestations of melasma‐like skin lesions in BALB/c mice. (a) Face group. (b) Head group. (c) Back group. *N* = 5 for each group, scale bar = 50 μM.

According to the draft [5], we selected different strains of mice, trying to establish a melasma‐like model by ultraviolet light combined with progesterone intramuscular injection and emotional stress recommended, to evaluate the sensitivity in modeling of different mice. On Day 28, the skin of the head, face, and back of KM and BALB/c mice in the control group was smooth, with no apparent erythema, desquamation, or texture changes (Figures 3 and 4). No apparent pigmentation was observed in the three regions of the body in the model group (Figures 3 and 4), and the skin of the irradiated regions on the face and head of both types of mice exhibited thickening and a leathery texture (Figures 3a,b and 4a,b). There was no significant change on the back skin of KM mice (Figure 3c). The back skin of BALB/c mice appeared uneven, exhibiting localized hyperplasia, atrophy, and thick wrinkles (Figure 4c). H&E staining revealed skin lesions in the model groups, showing slight local epidermal hyperplasia, a flattened epidermis–dermis junction, and obvious collagen proliferation in the dermis, with tightly arranged fibers (Figures 3 and 4). Further Masson‐Fontana staining revealed no melanin granules in the dermis under the microscope (Figures 3 and 4).

### 3.3 General Observation and Histological Manifestations of Melasma‐Like Skin Lesions in C57BL/6J Mice

**FIGURE 5:**
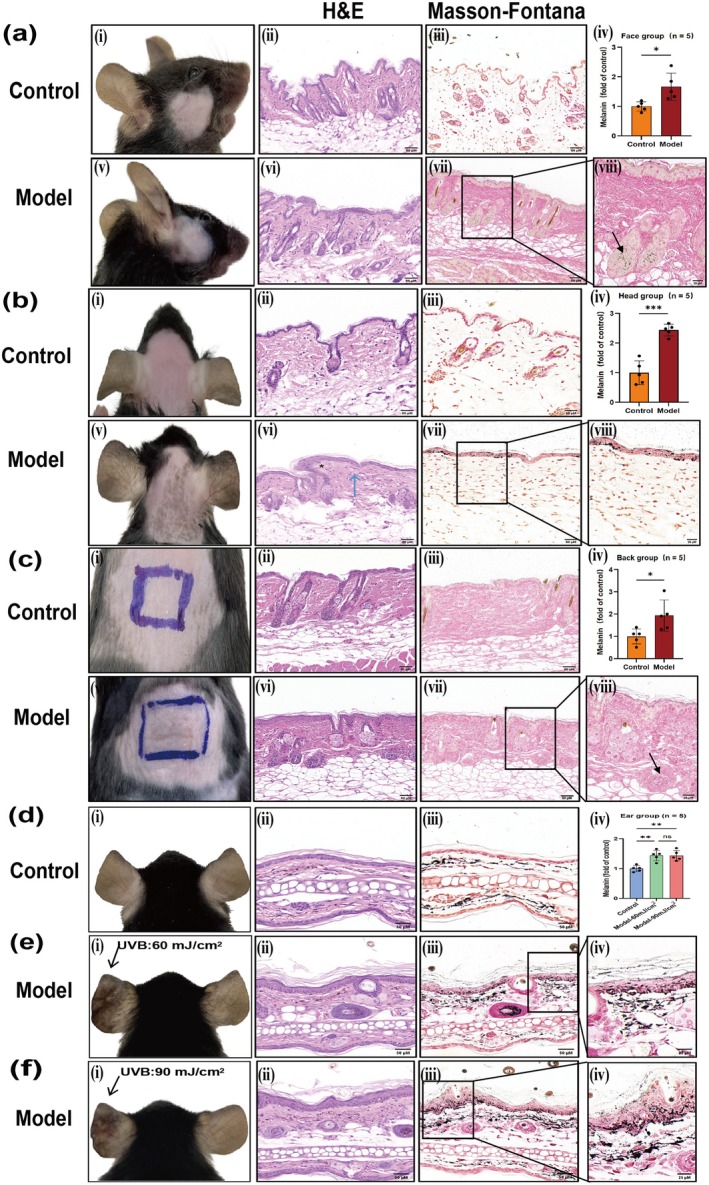
Clinical observation and histopathological manifestations of melasma‐like skin lesions of cheek, back and head in C57BL/6 mice. *N* = 5 for each group. Scale bar = 50 μM & 25 μM. Error bars, means ± SD. Welch's *t*‐test & One‐way ANOVA. *p < 0.05, **p < 0.01, ***p < 0.001. (a) Face group. →: increased melanin in hair follicle. (b) Back group. →: increased melanin in hair follicle. *The basal cells arrange disorderly in model group. →: pigmentophage in dermis in model group. (c) Head group. (d) Control group of ear. (e) Model group of ear with stimulating at 60 mJ/cm^2^. (f) Model group of ear with stimulating at 90 mJ/cm^2^.

Compared to the control groups, there were no obvious pigmentation spots on the face and back skin lesions, but the surface lost luster, was rough, with scattered desquamation and wrinkles in the model group (Figure 5a,c). Microscopic examination revealed slight epidermal hyperplasia, a flattened epidermis–dermis junction, and obvious collagen proliferation in the dermis, with tightly arranged fibers (Figure 5a,c). Further Masson‐Fontana staining demonstrated that melanin granules were diffusely distributed in the dermis (Figure 5a,c). The statistical analysis results indicated that the melanin content in the face and back was significantly higher compared to the control group (Figure 5a,c, *p* < 0.05). Still, we found that the increased melanin particles were mainly distributed in the hair follicles (Figure 5a,c).

The skin lesions on the head of C57BL/6J mice were evident as brown patches, with blurred boundaries and scattered central spot pigmentation (Figure 5b) on Day 28 compared to the control group. Under the microscope, the epidermis and dermis are scattered with melanin granules, the epidermis–dermis boundary is indistinct, liquefaction and degeneration of basal cells are observed, fusiform melanophages are present in the dermis, and collagen proliferation is noted, with close arrangement (Figure 5b). Masson‐Fontana staining showed that melanin granules were scattered throughout the entire epidermal layer, which was significantly higher compared to the control group (Figure 5b). The results of statistical analysis were consistent with the microscopic observations, and the melanin content in the head group was significantly higher than in the control group, being approximately three times greater than in the control group (Figure 5b, *p* < 0.001).

### 3.4 General Observation and Histological Manifestations of Melasma‐Like Skin Lesions in the Ear of C57BL/6J Mice

Modeling in the ear of mice is commonly reported in studies on pigmented diseases, such as post‐inflammatory hyperpigmentation. Therefore, an attempt was made to further establish an ear model in C57BL/6J mice. According to MED, we set up a 60 mJ/cm^2^ group. In addition, we set up a 90 mJ/cm^2^ group because if the exposure is higher than 90 mJ/cm^2^, the mouse ear will appear to collapse. Compared to the control group, pigmented spots appeared significantly in the left ear of the two groups of mice irradiated by UVB, and the higher the radiation dose, the more obvious the curl and thickening of the ear on Day 28 (Figure 5d–f). Under the microscope, it was observed that the epidermis of the model was significantly thickened, melanophages were observed in the superficial dermis, numerous melanin granules were distributed throughout the entire dermal layer, and collagen proliferation was noted, with close arrangement. Increased irradiation dose was associated with more pronounced epidermal hyperplasia and hyperkeratosis in the mouse ear (Figure 5d–f). Masson‐Fontana staining showed that melanin granules were scattered throughout the entire epidermal layer and were significantly more abundant compared to the control group (Figure 5d–f). Further statistical analysis showed that the melanin content of the model group was significantly higher than that of the control group (Figure 5d, *p* < 0. 01), but there was no significant difference among different irradiation doses (Figure 5d, *p* < 0.05). For the sake of reducing damage to the skin and the convenience of experimental operation, choosing a low radiation dose is recommended in modeling.

### 3.5 Comparison of General and Histological Features of Melasma Between Human and Melasma‐Like Mouse Model in C57BL/6J Mice

**FIGURE 6:**
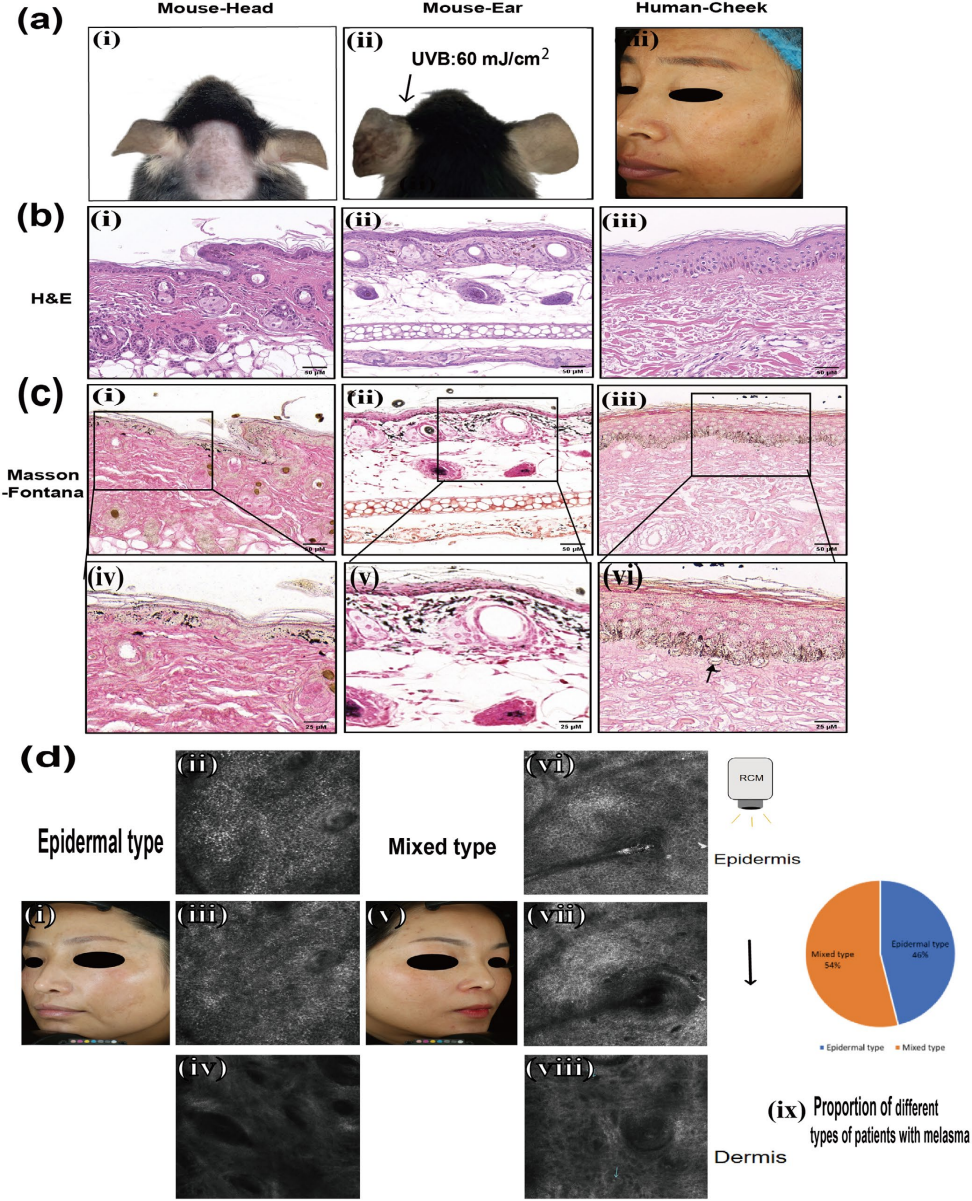
Clinical and histopathological features of melasma between human and melasma‐like mouse model in C57BL/6 mice. (a) General observation of skin lesions of melasma‐like mouse model in C57BL/6 mice and patient. (b) H&E staining results of different groups, scale bar = 50 μM. (c) Masson‐Fontana staining results of different groups. (i, iv) Head group; (ii, v) ear group; (iii, vi) patient with melasma. Scale bar = 50 μM & 25 μM.→: pendulous melanocytes.

A comparison was made between the skin lesions of patients with melasma and those of the melasma‐like mouse model in C57BL/6J mice. Skin lesions of the patient mainly manifested as irregular light brown patches with dotted pigmentation, and their boundary was indistinct (Figure 6a). Because obtaining human skin tissue is difficult, we attempted to detect more details of human melasma using RCM, a noninvasive imaging technique. We involved 26 patients with melasma. It was revealed that 46% (12/26) of patients were of the epidermal type, with a significant increase in melanin in the epidermis. Additionally, 54% (14/26) of patients were of the mixed type, with increased melanin observed in both the epidermis and dermis, as well as the presence of melanophages in the upper epidermis (Figure 6d).

In a melasma‐like mouse model, the skin lesions on the head and ears exhibited clinical manifestations similar to those observed in patients (Figure 6a). A significant increase in melanin granules was observed in the epidermis and dermis of patients, particularly in the epidermis, and pendulous melanocytes were clearly observed with Masson‐Fontana staining. In C57BL/6J mice, H&E and Masson‐Fontana staining of lesions demonstrated a significant increase in melanin particles in the epidermis and dermis of both the head and ears, with some melanin particles distributed in a band‐like pattern (Figure 6b,c).

### 3.6 Respones to TXA of Melasma‐Like Mouse Model

**FIGURE 7:**
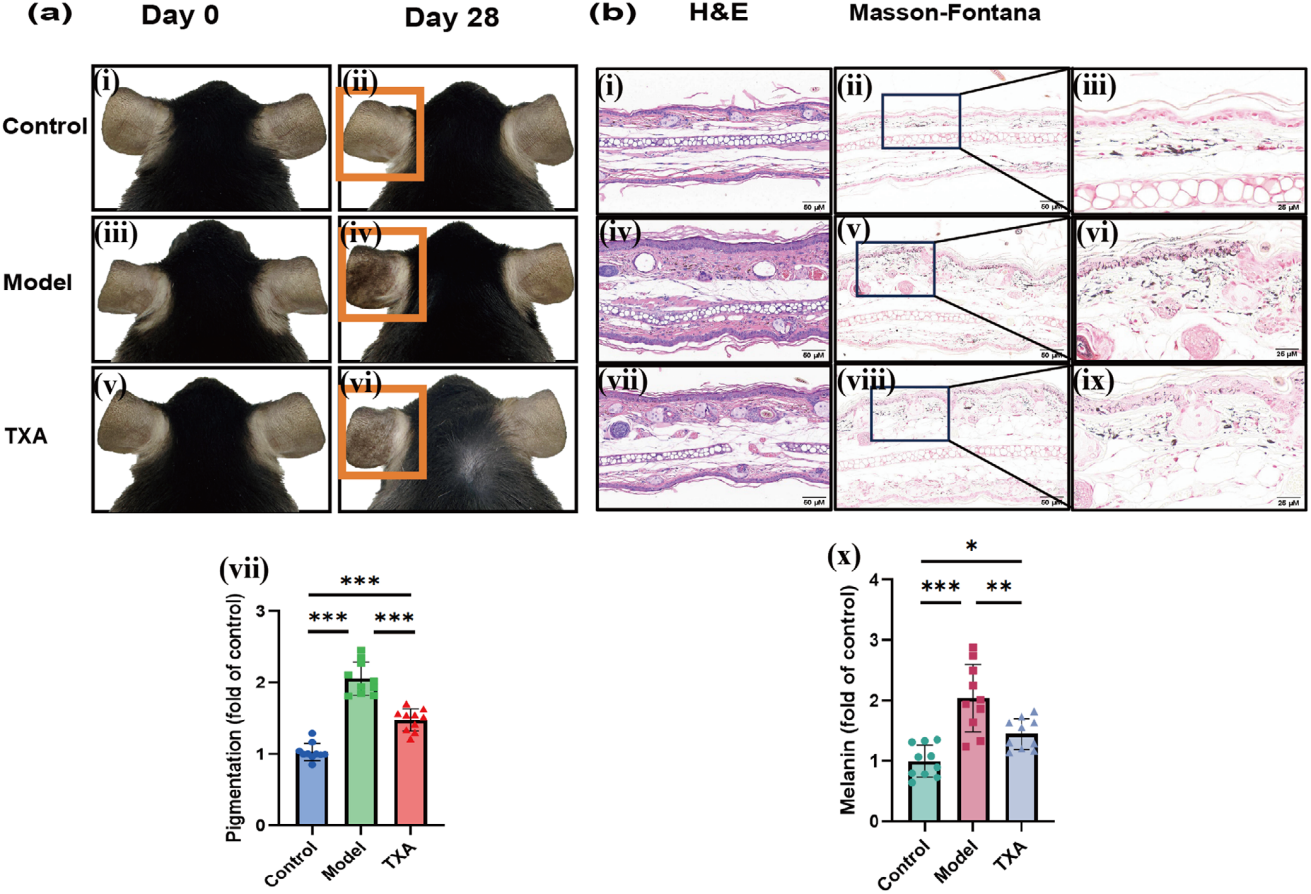
Respones to tranexamic acid of melasma‐like mouse model. (a) Clinical observation of different groups of mice. (b) H&E staining and Masson‐Fontana staining results of different groups of mice. *N* = 10 for each group. Scale bar = 50 and 25 μM. Error bars, means ± SD. Welch's *t*‐test & One‐way ANOVA. *p < 0.05, **p < 0.01, ***p < 0.001.

In order to validate the stability of the model and the potential of clinical drug evaluation, we expanded the sample size and selected TXA, a clinically recognized drug for melasma treatment, which was administered to the C57BL/6J melasma‐like mouse model. The results showed that the pigmentation of mice in the model group was significantly increased compared to the control group, while the pigmentation of mice in the TXA group was significantly decreased compared to the model group (Figure 7a). The results of H&E staining and Masson‐Fontana staining of lesions showed that the melanin content in the model group was significantly increased compared to the control group, while in the TXA group it was, on the contrary, significantly decreased compared to the model group (Figure 7b).

## 4 Discussion

Melasma is a symmetrical and irregular tan patch that often occurs in the exposed area (especially on the face), which often occurs in people with Fitzpatrick III–V type [11]. A suitable animal model is helpful to further clarify the pathogenesis and find better ways of prevention and treatment of melasma. At present, there is no recognized method of creating melasma‐like animal models all over the world. Our findings help to develop a sensitive, stable, and reproducible melasma‐like mouse model, enabling the evaluation of potential therapeutic strategies and facilitating the discovery of more effective treatments. Insights gained from these studies are anticipated to lead to improved management and prevention strategies for individuals affected by melasma.

Previously, it has been reported that albino mice such as KM and BALB/c were used to study pigmented diseases such as melasma [12, 13]. After modeling with KM and BALB/c mice for 28 days, we did not observe typical pigmented patches, while C57 BL/6 J mice showed the opposite with patches appearing, and the results of Masson‐Fontana staining were consistent with clinical observation. Owing to the reason known to all, KM and BALB/c mice are albino mice. Tyrosine kinase, which is the key enzyme of melanin synthesis, is mutated mainly by gene editing in KM and BALB/c mice [14]. Immunohistochemical staining for melan A and SOX 10 showed that melanocytes of these strains of mice are available; however, it is reported that they do not have the activity of synthesizing melanin granule [15, 16], so these strains of mice are not easy to stimulate pigmentation. C57 BL/6 J mice are easier to stimulate pigmentation for they could synthesize melanin normally. The typical clinical manifestation of melasma is irregular pigmentation, and active melanocytes are one of the important pathological features of melasma, so we think that albino mice are not the first choice for the establishment of a melasma‐like animal model.

It is reported that ultraviolet radiation plays a key role in the occurrence and development of melasma [1]. Currently, there are no authoritative standards for the dose, ranged from 120 to 166 mJ/cm^2^ per time [10, 17]. Research reports applying 2 ~ 3 MED in ultraviolet radiation [18], we did preexperiment of MED in different regions of mice, but after three times of irradiation with 2 ~ 3 MED, the skin of mice showed obvious inflammatory damage such as erythema, ulceration, and exudation. Therefore, we ultimately chose the current dose of irradiation to minimize local skin inflammatory damage in mice while inducing pigmentation. Additionally, research compared using only ultraviolet radiation and a combination of ultraviolet radiation and progesterone injection in vivo and showed that combined group can lead to abnormal protein expression in fatty acid and phospholipid metabolism, which may be closer to the environment of melasma formation [19]. Clinical studies reported 50% of pregnant women will develop melasma in the second and third trimesters [20]. These findings emphasized the importance of high progesterone levels in the pathogenesis of melasma. Further research reported anxiety traits is the risk factor of melasma [21]. In the research of traditional Chinese medicine, melasma is also known as “liver spot”. The most common cause is “liver stasis and qi stagnation”, while emotional abnormality and pressure are the leading causes of liver qi stasis. Therefore, our study combined UVB, progesterone injection and emotional stress to mimic the complex interactions of multiple factors that contribute to melasma in clinical practice.

It is widely known that there are two types of melasma—epidermal type and mixed type, based on the location of increased melanin [22]. The RCM findings in our study are consistent with previous reports. It has been reported that guinea pigs can be used to mimic melasma‐like conditions; however, the results showed that melanin granules were deposited only in the epidermis, indicating that guinea pigs can only simulate the epidermal type of melasma [19]. In our study, C57BL/6J mice were used to develop a melasma‐like model using the method described in this study. Masson‐Fontana staining of the lesion of our mouse model revealed the deposition of melanin granules in both the dermis and epidermis, mimicking the mixed type of melasma, which is consistent with the manifestations observed in some patients.

As melasma often occurs in the face, and the previously published studies mostly use the back to create the animal model, we picked the cheek, head, back, and ear of mice models. In modeling with C57BL/6J mice, typical pigmentation was not observed in the model group on the face and back. Masson‐Fontana staining showed that the distribution of scattered melanin particles in the dermis was higher than that in the control group, and the melanin content analysis showed that the melanin content in the dermis increased slightly. The sensitivity of different anatomical parts of the skin in mice to stimuli varies, which is consistent with clinical studies that the severity of pigmentation varies in different anatomical regions, including the face and upper limbs [23]. It suggests that the onset of melasma may be related to the mechanism based on histological differences at different locations. Meanwhile, it has been reported that the subsets of dermal fibroblasts vary in different anatomical parts of the human body, and they can participate in the pathogenesis of pigmentary diseases, such as vitiligo, by regulating the autoimmune response [24]. In addition, the density of sebaceous glands and vascular and skin barrier function vary in different parts of the skin [25]. It is reported that cytokines secreted by sebaceous gland cells can induce dendritic formation and melanin synthesis in melanocytes [26]. Besides, patients with melasma have abnormal barrier function and vascular distribution [27]. In our study, C57BL/6J mice exhibited a greater propensity for patch formation on their ears and heads than on their cheeks, while patients typically develop melasma on their faces, indicating differences in sensitive regions between the two species. Studies have reported that sebaceous glands are more densely distributed in the ears and heads of mice than in their cheeks [28], whereas sebaceous glands in the human face are more abundant and not covered by hair. This anatomical variation may partly explain the differences in sensitivity between species. However, this distinction requires further investigation.

In our study, a limited number of mice were utilized in each group, and the sample size will be further expanded to verify the stability of the melasma mouse model. Additionally, beyond the clinical manifestations of human melasma, other features include genetic susceptibility, high estrogen levels, a compromised skin barrier, altered oxidative stress levels, and abnormal expression of melanin synthesis and transport‐related enzymes in skin tissue. In this study, the strains, body regions, and dosages of mice were selected based solely on phenotypic characteristics, which represent a limitation of the study. Plans have been made to establish a model in accordance with the aforementioned methods to further investigate relevant molecular changes. Moreover, RCM, a noninvasive technique, was used to observe histological changes in the skin of patients with melasma, which may not fully reflect the actual physiological conditions.

In summary, an attempt was made to establish a melasma‐like animal model in various regions of mice using different strains. It was found that modeling in the head and ear regions of C57BL/6J mice was more consistent with the clinical manifestations of melasma in humans. Additionally, it is recommended to use a radiation dose of 30 mJ/cm^2^ for the head group and 60 mJ/cm^2^ for the ear group to study the pathogenesis, prevention, and treatment of melasma.

## Author Contributions

T.L., X.C., and Y.Y. designed, supervised, and directed the study; X.S. wrote the original draft; X.S., W.W., and H.Y. conducted experiments and analyzed data. T.L., X.C., Y.Y., H.Y., and Y.L. revised the draft. H.D., X.Z., X.L., S.T., and X.L. conducted experiments.

## Ethics Statement

All the animal experiments were approved by the Animal Ethics Committee of the Dermatology Hospital of the Chinese Academy of Medical Sciences and conformed to the existing current animal welfare guidelines (approval number: 2023‐DW‐014; the date of this approval: Oct. 25th, 2023). All mice were raised in accordance with the animal experimental guidelines of the Experimental Animal Center of the Dermatology Hospital of the Chinese Academy of Medical Sciences. All the studies of human volunteers were approved by the Ethics Committee of the Dermatology Hospital of the Chinese Academy of Medical Sciences (approval number: 2020‐KY‐011; the date of this approval: September 15, 2020) and (approval number: 2022‐K‐Y‐039; the date of this approval: August 19, 2022). This study was conducted in accordance with the principles of the Declaration of Helsinki (1964). Written informed consent was obtained from all participants before the commencement of the treatment.

## Conflicts of Interest

The authors declare no conflicts of interest.

## Data Availability

The data that support the findings of this study are available from the corresponding author upon reasonable request.
